# The influence of different transcatheter aortic valve implantation angles, as assessed by multidetector spiral CT scanning, on the depth of valve implantation and its impact on clinical prognosis

**DOI:** 10.3389/fmed.2026.1694319

**Published:** 2026-03-03

**Authors:** Shuyi Zeng, Xiangwen Liang

**Affiliations:** Department of Cardiology, Yulin First People’s Hospital, Yulin, China

**Keywords:** commissural alignment, complications, implantation depth, quality of life, transcatheter aortic valve implantation (TAVI)

## Abstract

**Purpose:**

This study aimed to investigate the impact of commissural alignment on transcatheter aortic valve implantation (TAVI) depth and clinical outcomes using multidetector spiral computed tomography (MSCT) assessment.

**Methods:**

In a retrospective cohort of 215 high-surgical-risk aortic stenosis (AS) patients undergoing TAVI (April 2020–March 2024), patients were stratified into Aligned (0–30° commissural offset, *n* = 106) and Misaligned (>30° offset, *n* = 109) groups based on pre/post-procedural MSCT. Primary endpoints included implantation depth, [left ventricular ejection fraction (LVEF)], 30-days complications, and quality of life [Toronto Aortic Stenosis Quality of Life questionnaire (TASQ)].

**Results:**

Misaligned patients exhibited lower implantation depths at non-coronary (7.15 ± 0.53 mm vs. 6.95 ± 0.81 mm, *p* = 0.035) and left coronary cusps (5.21 ± 0.64 mm vs. 4.92 ± 0.41 mm, *p* < 0.001), with higher rates of deep implantation (>10 mm: 9.17% vs. 1.89%, *p* = 0.020). Misalignment increased complications: paravalvular leakage (19.27% vs. 6.60%, *p* = 0.006), pacemaker implantation (11.93% vs. 3.77%, *p* = 0.027), and valve dislocation (15.60% vs. 5.66%, *p* = 0.018). Aligned patients reported better 1-month TASQ scores (76.65 ± 17.31 vs. 69.85 ± 16.41, *p* = 0.003), particularly in physical limitations (*p* = 0.004) and emotional impact (*p* = 0.013).

**Conclusion:**

Commissural misalignment was associated with deeper valve implantation, higher complication rates, and reduced early quality of life. Precise angular alignment could optimize TAVI outcomes, potentially by improving implantation depth consistency and reducing early adverse events.

## 1 Introduction

Aortic stenosis (AS) represents a critical and growing global health challenge, particularly among aging populations. Epidemiological studies reveal a striking age-dependent prevalence, affecting approximately 0.3% of adults aged 18–44 years, rising to 2%–7% in those aged 45–75 years, and reaching 11.7%–12.4% in individuals over 75 years (1). This progressive valvulopathy, predominantly driven by degenerative calcification (81.9% of cases), imposes severe clinical consequences including heart failure, syncope, and a precipitous decline in survival once symptomatic, with average life expectancy plummeting to 1–3 years without intervention (1, 2).

Transcatheter aortic valve implantation (TAVI) has revolutionized AS management since its first-in-human description in 2002 (3). Initially reserved for inoperable or high-surgical-risk patients, TAVI indications have expanded dramatically, now encompassing intermediate and even low-risk cohorts (4). This paradigm shift is supported by guidelines endorsing TAVI for high-risk inoperable patients (Class I) and intermediate-risk patients (Class IIa) (4). Technical refinements (including reduced delivery systems (14–16Fr), optimized access routes (predominantly transfemoral), and enhanced prosthesis designs) have significantly improved procedural safety and efficacy (4, 5). Nevertheless, persistent complications such as paravalvular leakage, conduction abnormalities requiring permanent pacemaker implantation, and coronary obstruction continue to impact long-term outcomes (4).

Preprocedural planning with multidetector computed tomography (MDCT) has become indispensable for mitigating these risks. MDCT enables precise annular sizing, assessment of coronary ostia height, quantification of calcification patterns, and identification of optimal fluoroscopic projection angles (6–8). Its integration into clinical workflows has reduced vascular complications to 4%–5% and improved valve sizing accuracy, directly correlating with reduced PVL (6, 8). Despite these advances, specific details of the procedure (particularly prosthesis implantation depth and commissural alignment) remain inadequately characterized regarding their impact on clinical prognosis. While suboptimal implantation depth is linked to PPI and PVL (9, 10), the influence of angular orientation on long-term outcomes is mechanistically plausible yet underexplored.

The relationship between the THV implantation angle (commissural alignment) and the achieved implantation depth, is not well characterized (11). It is plausible that the rotational position of the valve during deployment could influence its final longitudinal position within the annulus and LVOT, potentially due to asymmetric interactions with native anatomy or calcium distribution (12). MSCT, with its high spatial resolution and multiplanar reconstruction capabilities, provides the unique ability to precisely quantify both commissural alignment angles and post-procedural implantation depth in three dimensions (13). Understanding whether a specific relationship exists between these two positioning parameters could refine deployment strategies and enhance procedural planning (9).

Therefore, this study aimed to investigate the potential influence of different TAVI angles, as rigorously assessed by pre- and post-procedural MSCT, on the achieved depth of valve implantation. Furthermore, we explored the combined impact of implantation angle and depth on key clinical and hemodynamic outcomes following TAVI. With this study, we endeavor to provide deeper insights into the complex dynamics of THV positioning and its implications for optimizing patient prognosis after TAVI through detailed MSCT analysis.

## 2 Materials and methods

### 2.1 Case selection

A retrospective cohort analysis was conducted on 215 patients with AS who underwent TAVI at our hospital from April 2020 to March 2024. Data were collected through the medical record system, including patient demographics, implantation depth, and the incidence of postoperative complications. All procedures in this study adhered to the ethical guidelines established by the Declaration of Helsinki (1964 edition and subsequent revisions). In addition, the study received approval from the Institutional Review Board of the Yulin First People’s Hospital (Protocol number: YLSY-IRB-SR-2024023). Given that the data used in the study consisted of anonymized patient information and posed no potential risk or impact on patient care, the Institutional Review Board waived the requirement for informed consent.

### 2.2 Inclusion and exclusion criteria

Inclusion criteria: (1) the patient was 18 years old or older; (2) diagnosed with AS (14); (3) received TAVI and underwent multidetector spiral CT scanning; (4) all patients had an excessively high surgical risk and were deemed unsuitable for open-heart surgery; (5) normal cognitive function, able to cooperate with various treatments and examinations.

Exclusion criteria: (1) patients with a calculated glomerular filtration rate (GFR) of <35 mL/min; (2) known severe peripheral vascular disease; (3) patients with bicuspid aortic valves as such valves typically exhibit significant cusp asymmetry; (4) patients undergoing valve-in-TAVI procedures; (5) pregnant and lactating women. (6) Incomplete case records and follow-up data.

### 2.3 Grouping

Patients were divided into two groups based on different valve implantation angles: Aligned group and Misaligned group. 106 patients with commissural alignment were included in Aligned group, while 109 patients without commissural alignment were included in Misaligned group. Commissural alignment is defined according to the angular relationship between native and bioprosthetic valve commissures (15).

### 2.4 TAVI

The self-expanding Venus-A valve (Venus Medtech, China) and the self-expanding Vitaflow valve (MicroPort, China) were used. The primary approach through the femoral artery uses the pre-closure technique (Prostar XL, Abbott, USA) for femoral access. Direct puncture of the common femoral artery was ensured by iliofemoral angiography from the contralateral side. A single Prostar device was used for 18- to 19-F sheaths and 2 devices used for 22- and 24-F sheaths. The second Prostar device was placed at 45° to the first one. After Prostar deployment, the femoral artery introducer sheath was carefully inserted over a stiff guidewire. Following aortic valve deployment, the introducer sheath was retracted to the external iliac artery, and angiography was performed to assess for iliac artery complications. The femoral artery was subsequently closed by tying the Prostar sutures before a final iliofemoral angiogram was performed from the contralateral side. The procedural success and 30-days composite safety endpoint were assessed according to Valve Academic Research Consortium criteria.

In our center, commissural orientation was assessed and managed primarily through a standardized fluoroscopic deployment approach and post-procedural MSCT verification. During valve advancement and deployment, operators aimed to maintain a stable coaxial position in the annular plane and avoided excessive catheter torque once the delivery system crossed the native valve. Rotational orientation at the time of deployment was not guided by a dedicated manufacturer-specific alignment marker system, and no mandatory “commissural alignment” technique (e.g., pre-specified rotational target relative to a coronary cusp) was enforced throughout the study period. Therefore, the final commissural alignment observed on MSCT reflects real-world practice variability across operators and anatomies rather than assignment by protocol. This approach allowed comparison of clinical and imaging outcomes between patients who achieved commissural alignment versus those who did not.

### 2.5 MSCT image

Multidetector spiral computed tomography examinations were performed on a photon CT dual-source 64-slice CT (Somatom Definition FLASH, Siemens, Germany). A total of 120 ml of iohexol (JYHZ1700215, GE Healthcare, USA) was injected at a rate of 5 ml/s, followed by a 30 ml saline flush (G4702-500ML, Servicebio, China). The MSCT scanner detector collimation width was 0.625 mm, with a detector coverage of 40 mm, a reconstruction slice thickness of 1.25 mm, and a slice interval of 1.25 mm. The gantry rotation time was 0.35 s, and the pitch ranged from 0.16 to 0.20 (adjusted according to heart rate). All cases used ECG-gated dose modulation.

#### 2.5.1 Implantation angle

**FIGURE 1:**
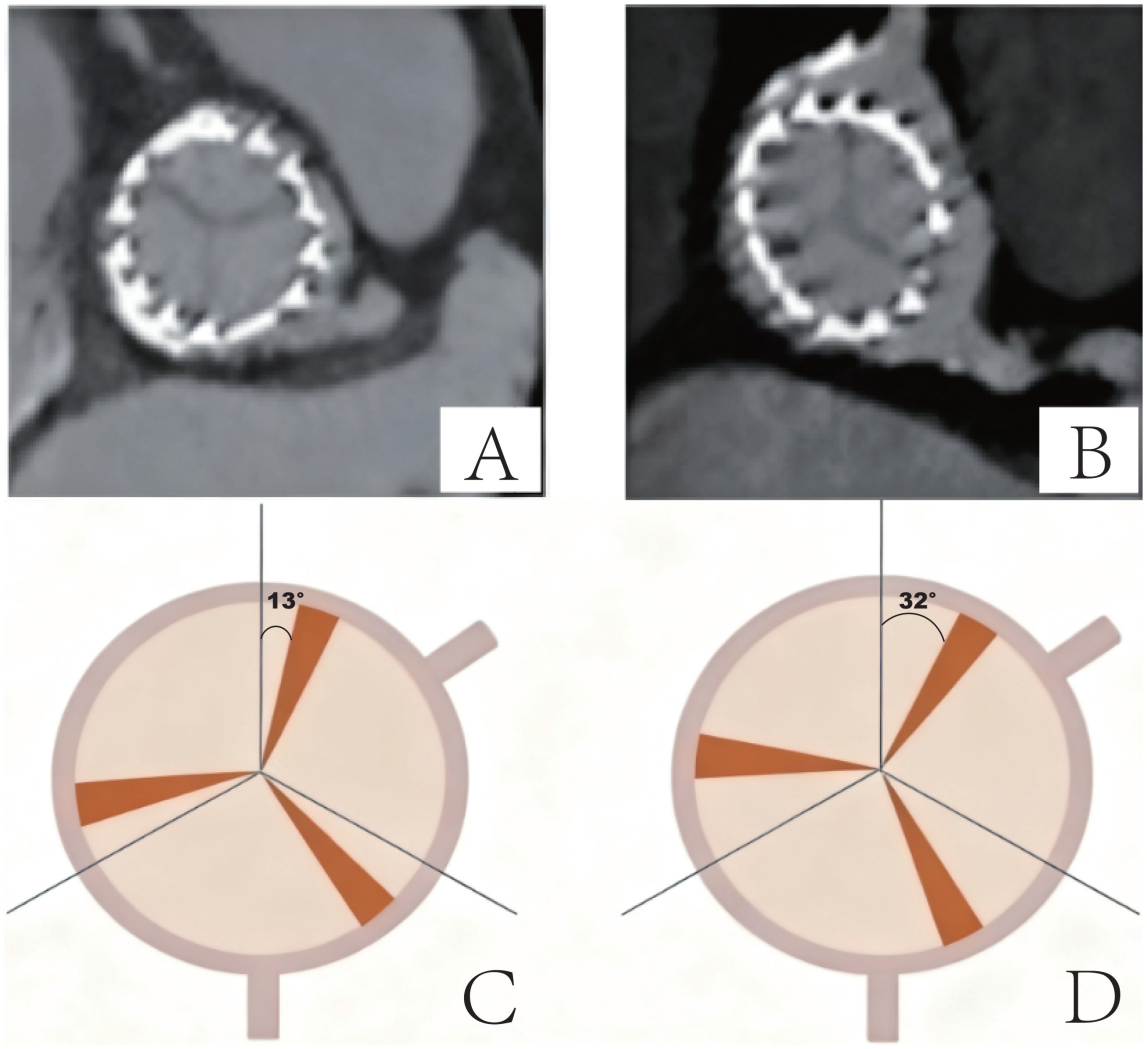
TAVR implantation angles - interstitial commissural alignment. **(A)** Aligned commissures - CT example (Δ≤ 30°); **(B)** Misaligned commissures - CT example (Δ > 30°); **(C)** Aligned orientation - schematic (Δ = 15°); **(D)** Misaligned orientation - schematic (Δ = 32°).

Commissural alignment was analyzed post-TAVR by an independent observer. Angles of the individual commissures (i.e., right to left coronary commissure, left to noncoronary commissure, and non- to right coronary commissure) were measured in degrees clockwise relative to the right coronary ostium (defined as 0°) during the end of diastole at the level of coaptation in an en face view for both native and neocommissures. Absolute values of rotational orientation were used for all analyses. Commissural alignment was categorized with respect to the differences in commissural orientation between native and neocommissures as aligned (0–30°; Figures 1A,C) or misaligned (>30°; Figures 1B,D) (16).

#### 2.5.2 Implantation depth

**FIGURE 2:**
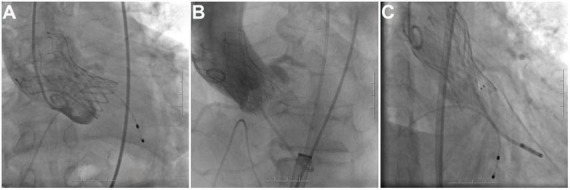
Implantation depth. **(A)** From the distal end of the prosthesis up to the nadir of the non-coronary cusp (NCC) and left coronary cusp (LCC); **(B)** standard implantation depth; **(C)** implantation position that is too deep.

Implantation depth was analyzed post-TAVR. To assess intra-observer and inter-observer variability, the ID was measured by one physician who was blinded to the outcomes. The implantation depth was measured by aortography in a coplanar view with the three native cusps aligned, assuring a coaxial frame position. In detail, the implantation depth was measured from the distal end of the prosthesis up to the nadir of the non-coronary cusp (NCC) and left coronary cusp (LCC). Valve oversizing was calculated as (prosthesis size − native annulus size/native annulus size) × 100. The optimal depth for prosthesis implantation is 4–10 mm below the aortic annulus, with a depth higher than 10 mm being considered as deep implantation (Figure 2).

Implantation depth was measured on MSCT by two physicians (blinded to clinical outcomes and group allocation) using a prespecified measurement protocol. To assess intra-observer variability, one reader repeated measurements in a randomly selected subset of cases after a washout period (≥2 weeks). To assess inter-observer variability, the second reader independently measured the same subset. Variability was summarized using intraclass correlation coefficients (ICC) and/or Bland–Altman analysis.

### 2.6 Follow up

Patients were evaluated 1 month after the treatment. The primary assessments included routine blood tests, incidence of complications, and quality of life. The 30-days mortality rate was also recorded. The observed complications in this study included valve dislocation, paravalvular leak, coronary artery obstruction, atrioventricular conduction block, and other clinical complications. Follow-up data were collected through outpatient or inpatient systems, records from external hospitals, and regular follow-ups. The follow-up cut-off date was January 10, 2025.

### 2.7 TASQ

Assessments using TASQ were conducted before treatment and 1 month after discharge. The Toronto Aortic Stenosis Quality of Life Questionnaire (TASQ) was used to assess the quality of life in patients with AS. It consists of a self-administered questionnaire with 16 items, encompassing four subscales: Physical Symptoms (2 items); Physical Limitations (4 items); Emotional Impact (7 items); Social Limitations (2 items) and Health Expectations (1 items). Participants are required to rate each item on a 7-point scale, ranging from “Not much” to “Very much.” Scores range from 16 to 112, with higher scores indicating a higher perceived quality of life. Cronbach’s alpha for the TASQ was 0.919 (17).

### 2.8 Statistical method

The chi-square test or Fisher exact probability method was used for the comparison of classified variables between two groups. For baseline comorbidities, each condition (e.g., COPD, PVD, prior PCI) was analyzed as an independent binary variable using χ^2^ or Fisher’s exact test, rather than as a single multi-category “past medical history” variable. A *t*-test or Mann-Whitney U test was used for the analysis of differences in the numerical variables between two groups. SPSS 20.0 and GraphPad Prism 5 statistical software were used for data analysis. All *p*-values were bilaterally distributed, and *p* < 0.05 indicated that the difference was statistically significant.

## 3 Results

### 3.1 Analysis of differences in general information of patients

No statistically significant differences were observed in demographic characteristics such as age, gender distribution, education level, place of residence, or smoking history between the Aligned and Misaligned groups (all *p* > 0.05, Table 1). Similarly, clinical characteristics including BMI, Logistic EuroSCORE, prevalence of hypertension, diabetes, stroke, positive family history, glomerular filtration rate, NYHA functional class distribution, and past medical history (prior MI, CABG, PVD, COPD, prior PCI) demonstrated no significant differences between the two groups (all *p* > 0.05, Table 2). As expected based on the predefined grouping criteria, the commissural offset angle was lower in the Aligned group (0–30°) and higher in the Misaligned group (>30°). Therefore, statistical comparison of commissural offset angle between groups is not clinically informative and is presented only to confirm correct group classification.

**TABLE 1:** Comparison of demographics characteristics between the two groups of patients.

Parameters	Misaligned group (*n* = 109)	Aligned group (*n* = 106)	t/χ ^2^	*p*
Age (years)	82.74 ± 6.65	82.86 ± 7.44	0.129	0.898
Gender [*n* (%)]			0.025	0.875
- Male	40 (36.70%)	40 (37.74%)		
- Female	69 (63.30%)	66 (62.26%)		
Residence [*n* (%)]			1.740	0.187
-Urban	80 (73.39%)	69 (65.09%)		
-Rural	29 (26.61%)	37 (34.91%)		
Smoking [*n* (%)]			0.100	0.951
-Never	35 (32.11%)	35 (33.02%)		
-Former	58 (53.21%)	57 (53.77%)		
-Current	16 (14.68%)	14 (13.21%)		

RSC, right sinus center; LROA, left-right sinus overlapping angle.

**TABLE 2:** Comparison of clinical characteristics between the two groups of patients.

Parameters	Misaligned group (*n* = 109)	Aligned group (*n* = 106)	t/χ ^2^	*p*
BMI (kg/m^2^)	27.82 ± 4.90	27.32 ± 4.34	0.805	0.422
Logistic EuroSCORE (%)	15.15 ± 3.37	15.41 ± 3.21	0.571	0.569
Hypertension [*n* (%)]	97 (88.99%)	94 (88.68%)	0.005	0.942
Diabetes [*n* (%)]	35 (32.11%)	38 (35.85%)	0.335	0.563
Stroke [*n* (%)]	7 (6.42%)	9 (8.49%)	0.334	0.563
Positive family history [*n* (%)]	21 (19.27%)	17 (16.04%)	0.385	0.535
Glomerular filtration rate (mL/min)	52.75 ± 7.81	51.24 ± 6.24	1.576	0.117
NYHA functional class [*n* (%)]			1.010 0.872	0.315 0.832
-I–II	8 (7.34%)	12 (11.32%)		
-III–IV	101 (92.66%)	94 (88.68%)		
Past medical history [*n* (%)]				
-PVD	2 (1.83%)	1 (0.94%)		
-COPD	19 (17.43%)	21 (19.81%)		
-Prior PCI	7 (6.42%)	9 (8.49%)		
-Without	81 (74.31%)	75 (70.75%)		
Commissural offset angle (°)	42.15 ± 5.07	18.14 ± 4.52	36.646	<0.001

BMI, body mass index; NYHA, New York heart association; MI, myocardial infarction; CABG, coronary artery bypass grafting; PVD, peripheral vascular disease; COPD, chronic obstructive pulmonary disease; PCI, percutaneous coronary intervention.

### 3.2 Comparison of aortic root between the two groups of patients

Analysis of aortic root parameters revealed that most measurements, including annular diameter, aortic valve area, LVOT diameter, SVC diameter, calcification volume, LCA height, RCA height, and STJ diameter, showed no significant differences between the Aligned and Misaligned groups (all *p* > 0.05, Table 3). However, significant differences were noted for aortic root angulation (52.93 ± 9.38° vs. 49.73 ± 10.46°, *t* = 2.363, *p* = 0.019), RSC prevalence (33.94% vs. 49.06%, χ^2^ = 5.059, *p* = 0.025), and LROA prevalence (59.63% vs. 42.45%, χ^2^ = 6.348, *p* = 0.012) (Table 3). These parameters were included to describe procedural viewing geometry and may reflect anatomical factors that influence the feasibility of achieving stable coaxial deployment and commissural alignment.

**TABLE 3:** Comparison of aortic root between the two groups of patients.

Parameters	Misaligned group (*n* = 109)	Aligned group (*n* = 106)	t	*p*
Annular diameter (mm)	27.34 ± 3.21	27.95 ± 2.93	1.454	0.147
Aortic valve area (cm^2^)	0.72 ± 0.22	0.75 ± 0.24	1.118	0.265
LVOT diameter (mm)	28.57 ± 3.82	28.05 ± 3.35	1.063	0.289
SVC diameter (mm)	33.06 ± 5.29	34.39 ± 4.83	1.928	0.055
Calcification volume (mm^3^)	575.63 ± 272.05	597.13 ± 212.36	0.647	0.518
Aortic root angulation (°)	52.93 ± 9.38	49.73 ± 10.46	2.363	0.019
LCA height (mm)	13.85 ± 3.59	14.75 ± 3.92	1.756	0.081
RCA height (mm)	16.62 ± 4.05	16.75 ± 3.13	0.278	0.781
STJ diameter (mm)	33.57 ± 3.92	33.32 ± 5.27	0.390	0.697
RSC [*n* (%)]	37 (33.94%)	52 (49.06%)	5.059	0.025
LROA [*n* (%)]	65 (59.63%)	45 (42.45%)	6.348	0.012

LVOT, left ventricular outflow tract; SVC, superior vena cava; LCA, left coronary artery; RCA, right coronary artery; STJ, sinotubular junction; RSC, right sinus center; LROA, left-right sinus overlapping angle.

### 3.3 Comparison of intraoperative conditions between the two groups of patients

Intraoperative conditions, including the size of the prosthesis implanted, preoperative peak pressure gradient, postoperative peak pressure gradient, and the distribution of surgical approaches utilized (transfemoral, transapical, subclavian, transaortic, transcervical), were comparable between the Aligned and Misaligned groups, with no statistically significant differences observed (all *p* > 0.05, Table 4). In addition, the distribution of prosthesis type (Venus-A vs. Vitaflow) was compared between groups, and no significant difference was observed, indicating that valve platform selection was unlikely to confound the relationship between commissural alignment and outcomes.

**TABLE 4:** Comparison of intraoperative conditions between the two groups of patients.

Parameters	Misaligned group (*n* = 109)	Aligned group (*n* = 106)	t/χ ^2^	*p*
Prosthesis size (mm)	28.31 ± 2.41	27.95 ± 1.57	1.323	0.187
Preoperative peak pressure gradient (mmHg)	59.27 ± 31.22	64.66 ± 38.83	1.119	0.265
Postoperative peak pressure gradient (mmHg)	5.45 ± 1.01	5.61 ± 1.26	1.037	0.301
Surgical approach [*n* (%)]			1.303	0.861
-Transfemoral	89 (81.65%)	81 (76.42%)		
-Transapical	11 (10.09%)	12 (11.32%)		
-Subclavian	5 (4.59%)	6 (5.66%)		
-Transaortic	3 (2.75%)	5 (4.72%)		
-Transcervical	1 (0.92%)	2 (1.89%)		

### 3.4 Comparison of implantation depth between the two groups of patients

While the mean overall implantation depth showed no significant difference between groups (*p* = 0.647, Table 5), specific measures revealed significant variations. The implantation depth relative to the NCC was significantly lower in the Misaligned group compared to the Aligned group (7.15 ± 0.53 mm vs. 6.95 ± 0.81 mm, *p* = 0.035). Similarly, the depth relative to the LCC was significantly lower in the Misaligned group (5.21 ± 0.64 mm vs. 4.92 ± 0.41 mm, *p* < 0.001). Consequently, the incidence of deep implantation (>10 mm) was significantly higher in the Misaligned group (9.17% vs. 1.89%, *p* = 0.020).

**TABLE 5:** Comparison of Implantation depth between the two groups of patients.

Parameters	Misaligned group (*n* = 109)	Aligned group (*n* = 106)	t/χ ^2^	*p*
Implantation depth mean (mm)	6.25 ± 2.11	6.12 ± 2.01	0.458	0.647
Implantation depth NCC (mm)	7.15 ± 0.53	6.95 ± 0.81	2.119	0.035
Implantation depth LCC (mm)	5.21 ± 0.64	4.92 ± 0.41	3.988	<0.001
Deep implantation [*n* (%)]	10 (9.17%)	2 (1.89%)	5.416	0.020

NCC, non-coronary cusp; LCC, left coronary cusp.

### 3.5 Comparison of quality of life between the two groups of patients

Quality of life, assessed using the TASQ questionnaire, showed significant benefits in the Aligned group at the 1-month follow-up. Patients in the Aligned group reported significantly better scores in Physical Limitations (18.65 ± 3.21 vs. 17.08 ± 4.52, *p* = 0.004), Emotional Impact (34.54 ± 6.31 vs. 32.52 ± 5.52, *p* = 0.013), and consequently, the TASQ Total Score (76.65 ± 17.31 vs. 69.85 ± 16.41, *p* = 0.003) compared to the Misaligned group (Table 6). No significant differences were observed in Physical Symptoms, Social Limitations, or Health Expectations subscales at 1 month, nor in any subscale pre-TAVI (*p* > 0.05).

**TABLE 6:** Comparison of TASQ between the two groups of patients.

Parameters	Misaligned group (*n* = 109)	Aligned group (*n* = 106)	t	*p*
**Physical symptoms (score)**				
-Pre-TAVI	6.46 ± 2.03	6.34 ± 3.33	0.318	0.751
-1 months after discharge	8.02 ± 2.03	8.45 ± 2.33	1.442	0.151
**Physical limitations (score)**				
-Pre-TAVI	12.23 ± 3.43	12.56 ± 4.41	0.609	0.543
-1 months after discharge	17.08 ± 4.52	18.65 ± 3.21	2.946	0.004
**Emotional impact (score)**				
-Pre-TAVI	30.24 ± 6.14	30.66 ± 5.14	0.546	0.585
-1 months after discharge	32.52 ± 5.52	34.54 ± 6.31	2.507	0.013
**Social limitations (score)**				
-Pre-TAVI	8.82 ± 4.32	8.73 ± 3.76	0.170	0.865
-1 months after discharge	9.01 ± 2.32	9.55 ± 1.76	1.932	0.055
**Health expectations (score)**				
-Pre-TAVI	2.48 ± 1.12	2.56 ± 1.01	0.566	0.572
-1 months after discharge	2.71 ± 1.07	2.76 ± 1.13	0.336	0.738
**TASQ total score**				
-Pre-TAVI	60.85 ± 21.97	61.65 ± 20.11	0.279	0.781
-1 months after discharge	69.85 ± 16.41	76.65 ± 17.31	2.955	0.003

TASQ, Toronto aortic stenosis quality of life questionnaire.

### 3.6 Comparison of complications and implantation success between the two groups of patients

**FIGURE 3:**
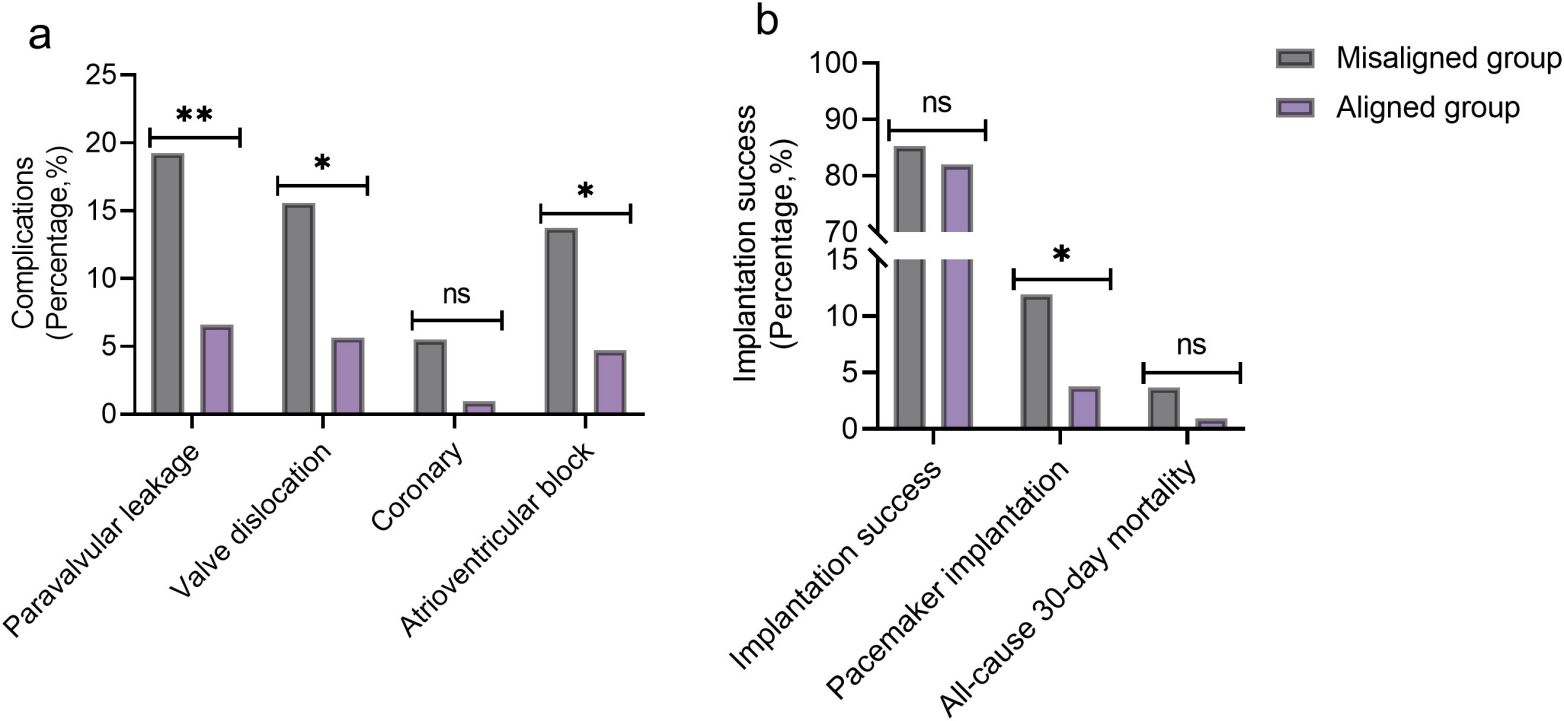
Comparison of complications and implantation success between the two groups of patients [*n* (%)]. **(a)** Complications; **(b)** implantation success. Ns, no significant; *P < 0.05; **P < 0.01.

The incidence of key procedural complications was significantly higher in the Misaligned group. This included Paravalvular Leakage (19.27% vs. 6.60%, *p* = 0.006), Valve Dislocation (15.60% vs. 5.66%, *p* = 0.018), and Atrioventricular Block requiring intervention (13.76% vs. 4.72%, *p* = 0.022) (Figure 3a). Coronary artery obstruction rates did not differ significantly (*p* = 0.134). Although overall implantation success rates at 6 months were comparable between groups (*p* = 0.519), the need for new permanent Pacemaker Implantation was significantly higher in the Misaligned group (11.93% vs. 3.77%, *p* = 0.027) (Figure 3b). All-cause 30-days mortality did not differ significantly (3.67% vs. 0.94%, *p* = 0.382).

## 4 Discussion

The present study provides evidence that commissural misalignment during TAVI, as assessed by pre- and post-procedural MDCT, is significantly associated with lower valve implantation depths, adverse hemodynamic sequelae, increased procedural complications, and reduced early quality of life. While the baseline characteristics, aortic root anatomy (except for increased aortic root angulation in the misaligned group), and intraoperative parameters were largely comparable between the aligned (0–30° commissural offset) and misaligned (>30° offset) groups, the divergent outcomes underscore the critical role of precise commissural alignment in optimizing TAVI results. Our findings extend beyond simple procedural metrics to encompass tangible functional and patient-centered benefits, offering mechanistic insights into the clinical impact of valve positioning.

### 4.1 Commissural alignment and implantation depth: an interdependent relationship

The observed lower implantation depths relative to the NCC and LCC in the misaligned group, alongside a significantly higher incidence of deep implantation (>10 mm), suggest a fundamental mechanical interaction between commissural orientation and final valve position. This phenomenon may be attributed to the geometric conflict arising when prosthetic commissures are significantly rotated relative to their native counterparts (18, 19). In a misaligned configuration, the prosthetic frame may encounter asymmetric resistance from the calcified native leaflets or annulus during deployment, potentially causing uneven expansion or downward migration (11, 20). Misalignment can alter the radial force distribution applied by the THV frame on the native aortic root complex. This change tends to promote deeper embedding of the valve to achieve a stable position (21). Our finding of an increased aortic root angulation in the misaligned group further supports this biomechanical hypothesis. A more horizontal aorta may amplify torsional forces during valve deployment and catheter manipulation, making precise commissural alignment technically more challenging and exacerbating the tendency for deep seating (11, 20). The specific increase in depth at the NCC and LCC locations, critical regions adjacent to the conduction system and mitral apparatus, provides a plausible anatomical link to the observed higher rates of conduction disturbances and paravalvular leak (18, 22).

### 4.2 Clinical outcomes: linking mechanics to morbidity

The significantly higher incidence of key complications in the misaligned group (paravalvular leakage, valve dislocation, and atrioventricular block requiring intervention) can be mechanistically linked to the observed lower implantation and potentially suboptimal valve expansion. Lower implantation, particularly at the NCC nadir, increases the proximity of the THV stent frame to the membranous septum and the atrioventricular conduction bundle, directly explaining the nearly threefold increase in pacemaker requirement (9, 23). Similarly, lower implantation can distort the annular sealing zone and increase the risk of PVL due to malapposition of the THV skirt (24, 25). Misalignment itself may exacerbate PVL risk by creating uneven gaps between the prosthetic leaflets and the native annulus where calcific nodules reside, hindering effective sealing, a concept supported by bench testing (24, 25). Valve dislocation risk is intrinsically linked to both depth (insufficient anchoring) and potentially asymmetric expansion caused by misalignment-induced uneven radial forces (26, 27). While coronary obstruction rates were not significantly different, the trend warrants caution, as misalignment could theoretically orient prosthetic leaflets toward a coronary ostium in vulnerable anatomies, an area needing further investigation (28, 29). Importantly, the significant improvement in quality of life (TASQ scores), particularly in physical limitations and emotional impact domains, in the aligned group at 1 month highlights the tangible patient benefit. This likely reflects the composite effect of fewer complications, and consequently, enhanced functional capacity and reduced procedural anxiety (30, 31).

### 4.3 Limitations and directions for future research

Some limitations of our study need to be considered. First, the retrospective design inherently carries risks of selection bias and unmeasured confounders, although the rigorous baseline matching mitigates this concern significantly. Second, the single-center nature and relatively short-term follow-up limit the generalizability and preclude assessment of long-term valve durability or late complications like structural valve deterioration potentially influenced by leaflet stress patterns related to alignment. Third, the use of a single THV type means the findings may not directly translate to self-expanding platforms, where alignment techniques and interactions with the native anatomy differ. It is also worth noting that while MDCT stands as the benchmark for evaluating alignment and depth, this study did not explore specific procedural elements that might influence alignment achievement, such as deployment techniques or imaging guidance protocols.

Future research endeavors should aim at addressing these limitations. There is a need for prospective studies involving multiple centers to confirm these results over extended periods, ideally lasting 5 years or more. Investigating the impact of commissural alignment on different types of valves, especially self-expanding ones, would provide valuable insights. Developing and accessing standardized deployment strategies to enhance alignment accuracy represents another crucial area of focus. Furthermore, understanding how alignment influences coronary access and valve longevity in the long run will be essential for improving clinical outcomes and patient care.

## 5 Conclusion

Commissural misalignment, as quantified by MSCT, was associated with deeper implantation at the NCC and LCC, higher rates of early complications (including PVL and pacemaker implantation), and lower 1-month quality-of-life scores. Given the retrospective observational design, these findings support a correlation between angular orientation and early post-TAVI outcomes rather than a proven causal relationship. Prospective multicenter studies using standardized commissural alignment techniques are warranted to determine whether actively optimizing rotational alignment can reproducibly improve implantation depth control and clinical prognosis.

## Data Availability

The original contributions presented in this study are included in this article/Supplementary material, further inquiries can be directed to the corresponding author.

## References

[1] Hamana T Sekimoto T Finn AV Virmani R. Age differences in aortic stenosis. *Rev Cardiovas Med.* (2025) 26:28185. 10.31083/RCM28185 40351685 PMC12059746

[2] Santangelo G Bursi F Faggiano A Moscardelli S Simeoli PS Guazzi M The global burden of valvular heart disease: from clinical epidemiology to management. *J Clin Med.* (2023) 12:2178. 10.3390/jcm12062178 36983180 PMC10054046

[3] Cribier A Eltchaninoff H Bash A Borenstein N Tron C Bauer F Percutaneous transcatheter implantation of an aortic valve prosthesis for calcific aortic stenosis: first human case description. *Circulation.* (2002) 106:3006–8. 10.1161/01.cir.0000047200.36165.b8 12473543

[4] Angioletti C Moretti G Manetti S Pastormerlo L Vainieri M Passino C. The evolution of TAVI performance overtime: an overview of systematic reviews. *BMC Cardiovas Disord.* (2024) 24:314. 10.1186/s12872-024-03980-2 38907344 PMC11191264

[5] Brankovic M Sharma A. Transcatheter aortic valve implantation and replacement: the latest advances and prospects. *J Clin Med.* (2025) 14:1844. 10.3390/jcm14061844 40142651 PMC11942769

[6] Okada A Beckmann E Rocher EE Fukui M Wang C Phichaphop A Preprocedural computed tomography planning for surgical aortic valve replacement. *Ann Thoracic Surg.* (2024) 117:1154–62. 10.1016/j.athoracsur.2024.02.017 38382704

[7] Abd Alamir M Nazir S Alani A Golub I Gilchrist IC Jr. Aslam F Multidetector computed tomography in transcatheter aortic valve replacement: an update on technological developments and clinical applications. *Exp Rev Cardiovas Therapy.* (2020) 18:709–22. 10.1080/14779072.2020.1837624 33063552

[8] Lopes V Almeida PC Moreira N Ferreira LA Teixeira R Donato P Computed tomography imaging in preprocedural planning of transcatheter valvular heart interventions. *Intern J Cardiovas Imag.* (2024) 40:1163–81. 10.1007/s10554-024-03140-9 38780710

[9] Baraka M Kamal D Mostafa AE. Depth of implantation in relation to membranous septum as a predictor of conduction disturbances after transcatheter aortic valve implantation. *Ind Pacing Electrophysiol J.* (2024) 24:133–9. 10.1016/j.ipej.2024.03.003 38548225 PMC11143730

[10] Eckel C Kim WK Schlüter J Renker M Bargon S Grothusen C Impact of accidental high or low implantation depth on peri-procedural outcomes after implantation with the self-expanding ACURATE neo2. *J Clin Med.* (2024) 13:5342. 10.3390/jcm13175342 39274553 PMC11396697

[11] Gorla R Oliva OA Arzuffi L Milani V Saitta S Squillace M Angulation and curvature of aortic landing zone affect implantation depth in transcatheter aortic valve implantation. *Sci Rep.* (2024) 14:10409. 10.1038/s41598-024-61084-5 38710782 PMC11074135

[12] Quagliana A Montarello NJ Willemen Y Bække PS Jørgensen TH De Backer O Commissural alignment and coronary access after transcatheter aortic valve replacement. *J Clin Med.* (2023) 12:2136. 10.3390/jcm12062136 36983139 PMC10056242

[13] Holzamer A Sitka E Hengstenberg C Schmid C Debl K Maier L Multislice computed tomography-based prediction of the implantation plane in transcatheter aortic valve implantation: determination of the line of perpendicularity and the implanter’s views. *Eur J Cardio-Thoracic Surg.* (2015) 48:879–85. 10.1093/ejcts/ezv095 25825262

[14] Grimard BH Safford RE Burns EL. Aortic stenosis: diagnosis and treatment. *Am Fam Phys.* (2016) 93:371–8. 26926974

[15] Tang GHL Amat-Santos IJ De Backer O Avvedimento M Redondo A Barbanti M Rationale, definitions, techniques, and outcomes of commissural alignment in TAVR: from the ALIGN-TAVR Consortium. *JACC Cardiovas Intervent.* (2022) 15:1497–518. 10.1016/j.jcin.2022.06.001 35926918

[16] Raschpichler M Flint N Yoon SH Kaewkes D Patel C Singh C Commissural alignment after balloon-expandable transcatheter aortic valve replacement is associated with improved hemodynamic outcomes. *JACC Cardiovas Intervent.* (2022) 15:1126–36. 10.1016/j.jcin.2022.04.006 35680192

[17] Świa̧toniowska-Lonc N Ściborski K Styra R Lüske CM Wêgrzynowska-Teodorczyk K Frank D Toronto Aortic Stenosis Quality of Life Questionnaire (TASQ): validation in polish patients with aortic stenosis. *J Clin Med.* (2025) 14:2502. 10.3390/jcm14072502 40217951 PMC11989317

[18] Ferri LA Ancona MB Papageorgiou C Vella C Capuano S Romano V Computed tomography derived predictors of left ventricular obstruction after TAVR. *Intern J Cardiol.* (2025) 422:132956. 10.1016/j.ijcard.2024.132956 39765320

[19] Mylotte D Fezzi S McInerney A. Standardising TAVI procedures: a step towards improved patient care. *Euro Intervent.* (2023) 19:e107–9. 10.4244/eij-e-23-00021 37283131 PMC10240726

[20] Breitbart P Czerny M Minners J Schröfel H Neumann FJ Ruile P. Impact of the aortic geometry on TAVI prosthesis positioning using self-expanding valves. *J Clin Med.* (2022) 11:2259. 10.3390/jcm11082259 35456350 PMC9025818

[21] Egron S Fujita B Gullón L Pott D Schmitz-Rode T Ensminger S Radial force: an underestimated parameter in oversizing transcatheter aortic valve replacement prostheses: in vitro analysis with five commercialized valves. *ASAIO J.* (2018) 64:536–43. 10.1097/MAT.0000000000000659 28885378

[22] Asif N Ayoade P Razzouk J Bohen D Tooker M Gladstone L Multilayer perceptron neural network analysis of fluoroscopic working angle on transcatheter aortic valve implantation complications. *Cureus.* (2024) 16:e59144. 10.7759/cureus.59144 38803728 PMC11129667

[23] Murray MK Hofmann E De Rosa R Mas-Peiro S Seppelt P Walther T Life beyond 5 Years after TAVI: patients’ perceived health status and long-term outcome after transcatheter aortic valve implantation. *J Intervent Cardiol.* (2019) 2019:4292987. 10.1155/2019/4292987 31772530 PMC6794985

[24] Sá MP Bloom JP Osho AA. Paravalvular leak after transcatheter aortic valve implantation: importance of preprocedural variables and intraprocedural assessment. *J Am Heart Assoc.* (2024) 13:e037850. 10.1161/jaha.124.037850 39268674 PMC11935608

[25] Pardo Sanz A Zamorano Gómez JL. How to improve patient outcomes following TAVI in 2024? recent advances. *Kardiol Polska.* (2024) 82:696–701. 10.33963/v.phj.101399 38973443

[26] Grubb KJ Tom SK Sultan I Sá MP. Overcoming prosthesis-patient mismatch with transcatheter aortic valve replacement. *Ann Cardiothoracic Surg.* (2024) 13:236–43. 10.21037/acs-2024-aae-27 38841088 PMC11148752

[27] Fukui M Bapat VN Garcia S Dworak MW Hashimoto G Sato H Deformation of transcatheter aortic valve prostheses: implications for hypoattenuating leaflet thickening and clinical outcomes. *Circulation.* (2022) 146:480–93. 10.1161/circulationaha.121.058339 35862182

[28] Ribeiro HB Rodés-Cabau J Blanke P Leipsic J Kwan Park J Bapat V Incidence, predictors, and clinical outcomes of coronary obstruction following transcatheter aortic valve replacement for degenerative bioprosthetic surgical valves: insights from the VIVID registry. *Eur Heart J.* (2018) 39:687–95. 10.1093/eurheartj/ehx455 29020413

[29] Garcia S Kereiakes DJ. Coronary obstruction after transcatheter aortic valve replacement: from risk prediction to prevention. *J Soc Cardiovas Angiography Intervent.* (2022) 1:100386. 10.1016/j.jscai.2022.100386 39132852 PMC11308054

[30] De Ronde-Tillmans MJ de Jager TA Goudzwaard JA El Faquir N van Mieghem NM Zijlstra F Long-term follow-up of quality of life in high-risk patients undergoing transcatheter aortic valve implantation for symptomatic aortic valve stenosis. *J Geriatric Cardiol.* (2018) 15:261–7. 10.11909/j.issn.1671-5411.2018.04.003 29915615 PMC5997614

[31] Zelis JM van ’t Veer M Houterman S Pijls NHJ Tonino PAL Netherlands Heart Registration Transcatheter Heart valve Implantation Registration Committee. Survival and quality of life after transcatheter aortic valve implantation relative to the general population. *Intern J Cardiol Heart Vasculature.* (2020) 28:100536. 10.1016/j.ijcha.2020.100536 32478166 PMC7251765

